# Towards non-invasive characterisation of coronary stent re-endothelialisation – An in-vitro, electrical impedance study

**DOI:** 10.1371/journal.pone.0206758

**Published:** 2018-11-05

**Authors:** Ian Holland, Christopher McCormick, Patricia Connolly

**Affiliations:** Department of Biomedical Engineering, University of Strathclyde, Glasgow, Scotland, United Kingdom; Medical Collge of Gerogia at Augusta University, UNITED STATES

## Abstract

The permanent implantation of a stent has become the most common method for ameliorating coronary artery narrowing arising from atherosclerosis. Following the procedure, optimal arterial wall healing is characterised by the complete regrowth of an Endothelial Cell monolayer over the exposed stent surface and surrounding tissue, thereby reducing the risk of thrombosis. However, excessive proliferation of Smooth Muscle Cells, within the artery wall can lead to unwanted renarrowing of the vessel lumen. Current imaging techniques are unable to adequately identify re-endothelialisation, and it has previously been reported that the stent itself could be used as an electrode in combination with electrical impedance spectroscopic techniques to monitor the post-stenting recovery phase. The utility of such a device will be determined by its ability to characterise between vascular cell types. Here we present *in-vitro* impedance spectroscopy measurements of pulmonary artery porcine Endothelial Cells, Human Umbilical Vein Endothelial Cells and coronary artery porcine Smooth Muscle Cells grown to confluence over platinum black electrodes in clinically relevant populations. These measurements were obtained, using a bespoke impedance spectroscopy system that autonomously performed impedance sweeps in the 1kHz to 100kHz frequency range. Analysis of the reactance component of impedance revealed distinct frequency dependent profiles for each cell type with post confluence reactance declines in Endothelial Cell populations that have not been previously reported. Such profiles provide a means of non-invasively characterising between the cell types and give an indication that impedance spectroscopic techniques may enable the non-invasive characterisation of the arterial response to stent placement.

## Introduction

Coronary artery disease is a leading cause of morbidity throughout Europe and worldwide [1]. The growth of an atherosclerotic lesion within an artery occludes blood flow and provides a site for thrombus formation. Treatment of the disease has progressed significantly over the recent decades and has been greatly aided by the development of an array of amelioratory intervention procedures of which coronary stent implantation can be considered one of the most successful. Since their first reported use in 1986 [2], coronary stents have moved through several phases of development, from first generation, bare metal versions to current modern drug eluting-devices [3].

Improvements in stent design have been driven by the need to reduce the post procedural occurrences of In-Stent Restenosis (ISR) and thrombosis. ISR is classified through angiography as a 50% reduction in the diameter of the artery in the region of stent implantation, through neointimal cell proliferation, inhibiting blood flow and endangering the life of the patient [4–6]. It is a consequence of damage to the arterial wall caused by stent expansion. The resulting inflammatory response to this vascular injury is a complex cascade mechanism that ultimately results in the proliferation of Smooth Muscle Cells (SMCs) over the stent struts. The advent of a class of stents with an anti-proliferative drug-eluting coating significantly reduced ISR occurrence and the most extensively used stents now present with ISR rates of around 3–4%, per year [7,8]. More recently neoatherosclerosis, the reoccurrence of lipid laden lesions, has been also been identified as an additional, later stage stent failure mode [9,10].

The optimal course of cellular regrowth following stent implantation is the reformation of a complete monolayer of functional endothelial cells (ECs) forming a barrier between the implant surface and the circulating blood [11,12]. Uncovered, exposed stent surfaces increase the risk of platelet activation, initiation of the coagulation cascade and the dangerous scenario of thrombus development [12]. To reduce the risk of thrombus formation, prolonged anti-platelet drug regimens are common. However, these come with an increased risk of bleeding and are not be suitable for all patients, particularly those with comorbidities [13,14]. With endothelialisation complete, withdrawal of anti-platelet therapy can be considered, however there are still no clinically available means of assessing the extent of EC proliferation over the stent struts. Current diagnostic techniques such as angiography, Magnetic Resonance Imaging and Computed Tomography have insufficient resolutions, as well as the presence of stent induced artefacts [15,16]. Consequently, these methods are unable to determine the status of the EC layer. Imaging modalities, such as optical coherence tomography and intravascular ultrasound, delivered to the stent region by catheterisation, can provide more detailed images showing cellular regrowth but are also limited in resolution and their invasive nature precludes routine use. Methods of non-invasively monitoring successful re-endothelialisation may address this limitation [17,18], and reveal wider insights into the mechanisms driving ISR and neoatherosclerosis.

A self-reporting stent, capable of providing temporal data on the vascular response to stent injury has been proposed, whereby the conductive properties of a metallic stent are exploited to carry out electrical impedance spectroscopic measurements to monitor cellular regrowth [18]. The concept of remotely monitoring neointimal growth in this way has been explored using mathematical modelling [19], an ex-vivo model [18], in-vivo balloon mounted electrodes [20], and patent activity in this area indicating possible future commercial developments [21,22]. The closest technological embodiment to this concept has been achieved by Oxley et al with an impedance-based stent, termed Stentrode. The device comprised a modified endovascular stent with platinum disk electrodes that recorded impedance variation via percutaneous leads that were implanted into sheep cerebral veins. The group identified an increase in capacitance that was attributed to neointimal growth [23,24]. However, the groups focus in developing such a device was as a step towards the recording and stimulation of neural activity and the vascular cells responsible for this impedance behaviour were not determined. Given the quite different roles that endothelial and smooth muscle cells play in clinical outcomes following stenting described above, it is therefore important to determine if impedance spectroscopy can non-invasively characterise between the vascular cell types associated with ISR and reendothelialization.

In-vitro use of impedance spectroscopy has been extensively used to non-invasively gain insights into a wide range of cellular behaviours such as micromotion [25], mitochondrial dysfunction [26], stem cell differentiation [27–29] and EC barrier function [30,31]. This research has been greatly facilitated by the availability of commercial impedance systems such as Applied Biophysics, ECIS system and ACEA Biosciences xCELLigence range. However, these systems feature small electrode geometries and are limited by the available frequency range resolution, with the xCELLigence system constrained to measurements at one of only 3 frequencies [31]. Cellular regrowth such as re-endothelialisation occurs over a range of exposed strut areas of 10-100mm2, several orders of magnitude greater than the micro scale electrodes commonly used in impedance studies. For the purposes of addressing the feasibility of a self-reporting stent to determine re-endothelialisation the electrode should be of a comparable size to capture data from a clinically relevant cell population.

Impedance measurements have previously been shown to be able to characterise different cell types in the determination of stem cell differentiation into osteogenic [27], adipogenic [28], neuronal [32] and cardiomyocyte [33] lineages. Haas et al, also demonstrated that the switching of rat aortic SMCs from a proliferative to a quiescent phenotype could be determined by reductions in total impedance measured across a 1kHz to 1MHz range [34]. Rümenapp et al, also reported distinct total impedance ratio variations across the same frequency range when investigating the effect of drug toxicity on murine fibroblasts [35]. We therefore hypothesised that impedance measurements in the high frequency kilohertz range would enable non-invasive characterisation between different vascular cell types as they proliferate to confluence.

To achieve the dual aims of wide frequency range measurement on stent comparable vascular cell populations we constructed a bespoke system capable of autonomously capturing impedance data in a physiologically relevant environment. Here we present the use of the developed system to carry out, impedance measurements of the primary vascular cell types pulmonary artery porcine endothelial cells, Human Umbilical Vein Endothelial Cells (HUVECs) and coronary artery porcine smooth muscle cells, as they proliferate to confluence.

## Materials and methods

### Cell culture chambers

**Fig 1:**
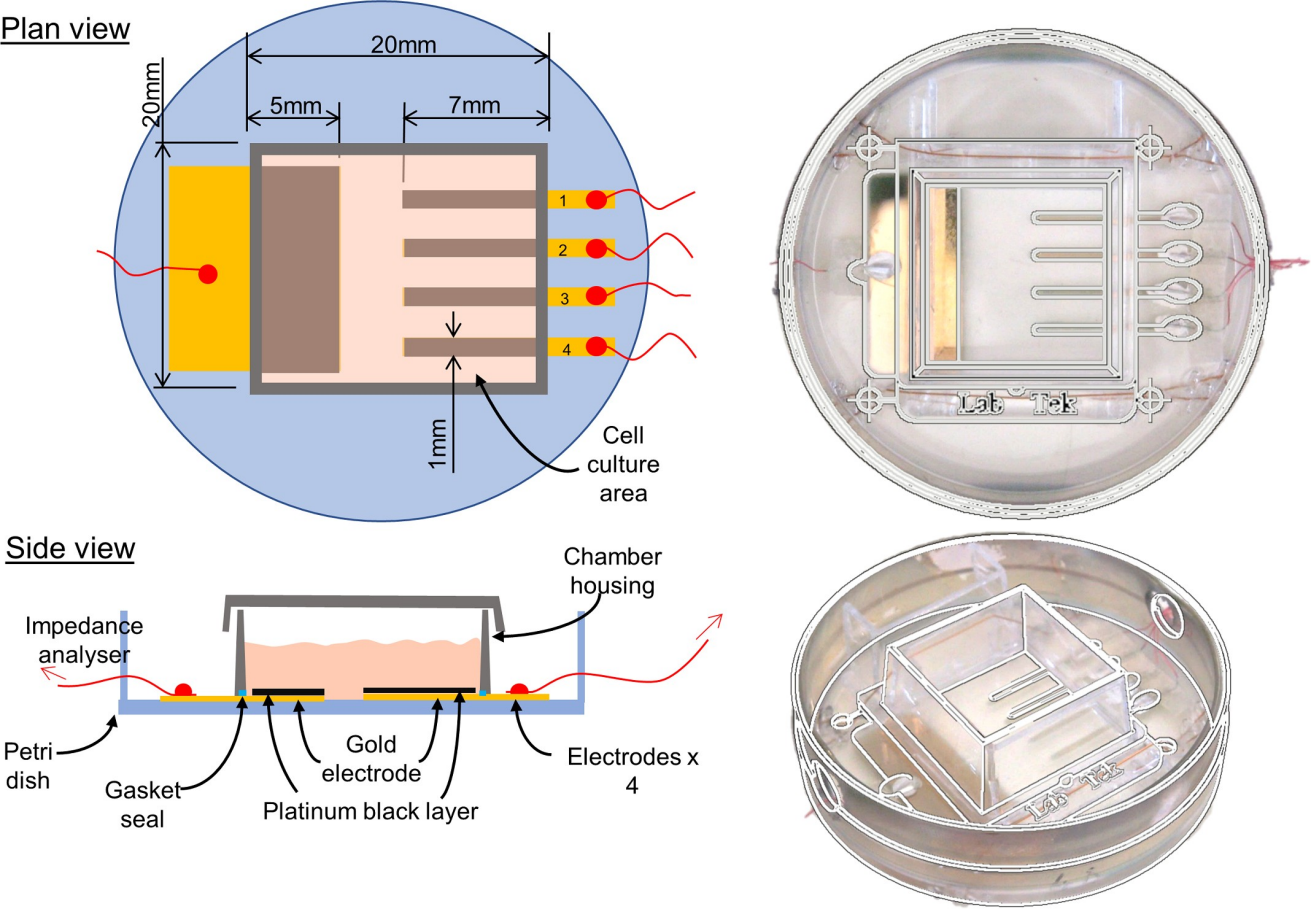
Chamber design with cell culture surface and electrode arrangement.

Gold electrodes were evaporated onto Falcon 35mm diameter petri dish [36] surfaces using the laser cut mask pattern shown in Fig 1. Compression of a 4.2cm2 culture cube (Lab-Tek chamber slide, Nunc, USA) onto the dish surface sealed a cell culture area containing a large reference electrode (100mm2) and an array of 4 smaller working electrodes (each 7mm2). Electrodes were galvanostatically coated with platinum black (36mA/cm2 for 1.5 seconds) in a 2 electrode setup with a platinum wire anode in platinising solution (0.06M Chloroplatinic acid hexahydrate and 794μM of lead acetate in deionised water, both, Sigma-Aldrich, Poole, United Kingdom) using a Solartron SI-1287 Electrochemical interface (Solartron Analytical, Farnborough, United Kingdom).

## Impedance measurement system

Impedance measurements at 50mV excitation voltage in the frequency range 1kHz to 100kHz were taken at a temporal resolution of 2 hours using a bespoke automated impedance system developed for this study. This allowed all measurements to be taken without removal of the cell culture chambers from the incubator. The system comprised an Autolab PGSTAT302N, (Metrohm, Herisau, Switzerland) with automated switching between 8 individual chambers performed using a 32 channel multiplexer (Analog devices, Norwood, Massachusetts, United States) controlled via an Arduino 2560 development board and a bespoke LabView program (National Instruments, Texas, United States). Impedance sweep data were logged as text files before chronological ordering and importation into Microsoft excel software.

### Cell culture

Primary ECs were isolated from the pulmonary arteries of pig hearts obtained fresh from a local abattoir. A scalpel blade was gently scraped across the luminal surface of a section of the pulmonary artery proximal to the heart. The cells were then suspended and cultured in Medium 200 with 2% Low Serum Growth Supplement (Invitrogen, Paisley, Scotland) and 1% penicillin–streptomycin (Sigma-Aldrich, Poole, United Kingdom). Primary SMCs were isolated from the left anterior descending coronary artery (LAD) from the same hearts, based on a previously optimised laboratory explant method [37]. Briefly, a 3cm section of the proximal LAD was dissected out from the heart and cut free from surrounding fat and connective tissue. The artery was then sectioned into 3mm length rings, which were transferred to cell culture flasks (2 rings per 25 cm2) containing 50:50 Waymouth's MB752/1 and F-12 Nutrient Mixture (Ham) (Invitrogen), 10% FBS (European source) and 1% penicillin–streptomycin. The artery rings were removed from the culture flask once it was clear that a viable population of cells with an SMC morphology had grown out from the artery and adhered to the culture surface. Typically, this took place around 10 days following the initial incubation. HUVECs, (Lonza, Basel, Switzerland, No. CC-2517) were grown in endothelial cell growth medium EGM2 BulletKit media (Lonza). All cell types were cultured in a humified atmosphere at 37°C (5% CO2/95% air). For impedance experiments cells were seeded into chambers at passages 2 to 4 at sub confluent densities to allow proliferation to confluence to be measured and observed. ECs were seeded into chambers in the density range 4.5–6.5 x 104 cells cm-2, SMCs 2.5–3.125 x 104 cells cm-2, HUVECs 4.125–5.1 x 104 cells cm-2, with no cells seeded into control chambers. Media was replaced every 48 hours in all chambers.

### Light microscopy

Electrodes were sufficiently transparent to permit imaging using an inverted light microscope (Motic, China) and cell growth to confluency was observed, with chambers removed from the incubator and images captured every 24 hours.

### Immunofluorescence

The primary porcine cell line isolation procedure was characterised initially with light microscopy observation of EC and SMC morphologies and subsequently through immunofluorescence imaging of paraformaldehyde fixed cells using Von Willebrand Factor (VWF) (1:400, Abcam, Cambridge, United Kingdom) for ECs and α Smooth Muscle Actin (αSMA) (1:400, Sigma-Aldrich,) for SMCs. Nuclei were stained with 4’,6-diamidine-2-phenylindole (DAPI) (1μg/ml, Sigma-Aldrich). Negative controls were performed by staining ECs for αSMA and SMCs for VWF. Fluorescent microscopy images were captured using a Zeiss Axio Imager Z1 (Cambridge, United Kingdom).

### Data presentation

For each cell type and control, impedance data were collated from electrodes and a mean calculated for each frequency at a given time point in the experiment. No cell-electrode circuit model was used to further process data presented in this paper. The maximum Standard Error Mean (SEM) is stated in the figure caption where error bars are not visible at the scales used. Three-dimensional representation of impedance data variation with time and frequency was carried out using dedicated LabView software.

## Results

### Immunofluorescence

**Fig 2:**
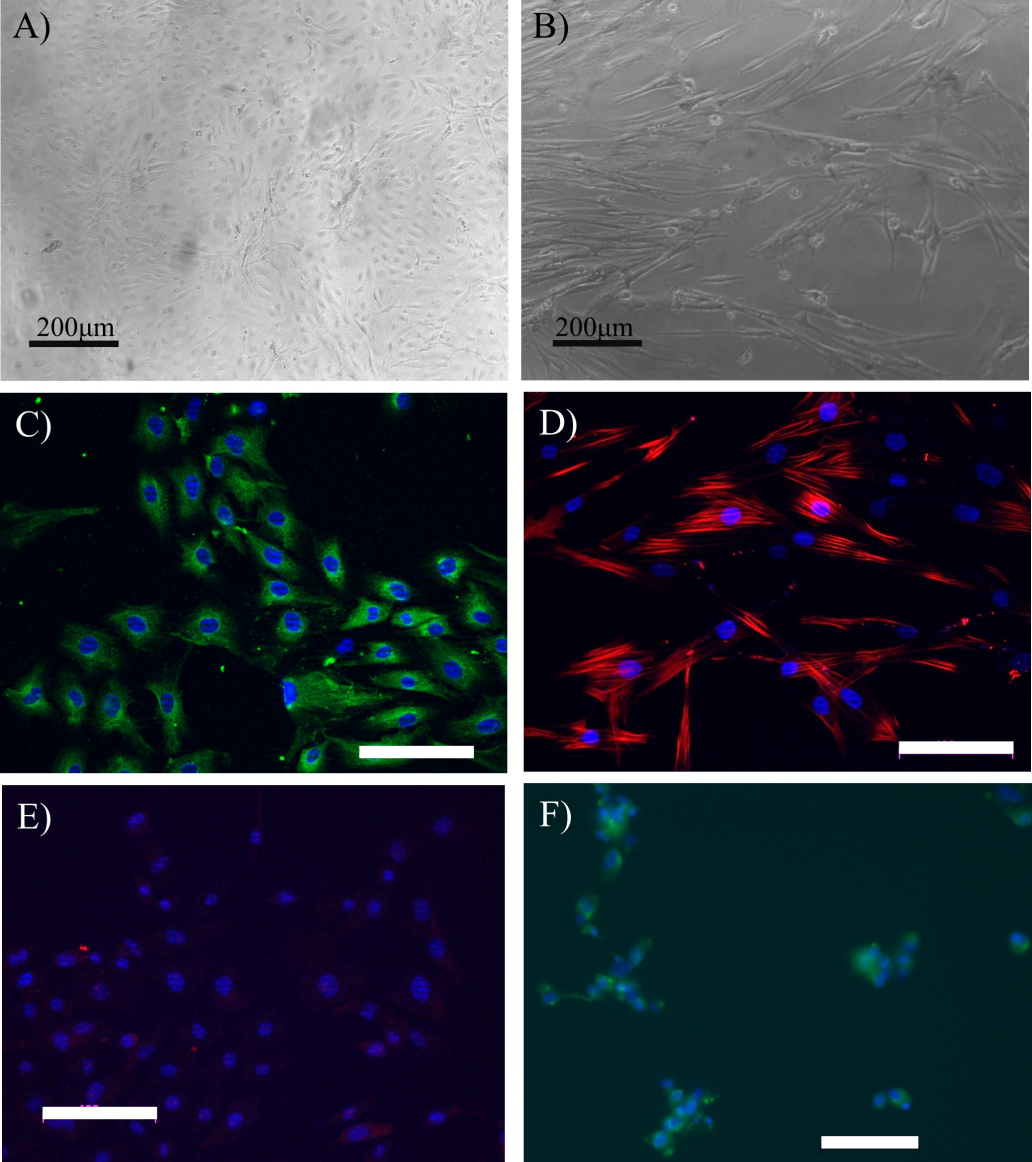
Primary cell isolation light microscopy and immunofluorescence characterisation. Light microscopy images showing typical morphologies for (A) isolated ECs and (B) SMCs. (C) ECs positively stained for the marker Von Willebrand factor (green). (D) Smooth muscle cells positively stained for alpha smooth muscle actin (red). (E) Negative control, ECs showing minimal staining for alpha smooth muscle actin (red). (F) Negative control, SMCs showing minimal staining for Von Willebrand factor (green). Images were taken of cells at passage 2. White scale bars are 100μm, all nuclei stained with DAPI (blue).

Successful isolation and establishment of EC and SMC lines was confirmed with immunofluorescence and light microscopy imaging. ECs in culture displayed the typical cobblestone morphology that is a well-established feature for this cell type [38,39] (Fig 2A) and expressed the endothelial cell marker VWF (Fig 2C). SMCs in culture displayed the elongated spindle morphology typical of smooth muscle cells [40] (Fig 2B) and expressed the smooth muscle cell marker αSMA (Fig 2D). Negative controls of ECs stained with αSMA showed no expression (Fig 2E). SMCs stained for VWF expression exhibited minimal staining indicating that a low level of ECs may be present in the isolated cell population as previously reported for SMC isolation protocols [41] (Fig 2F).

### Electrode platinisation

**Fig 3:**
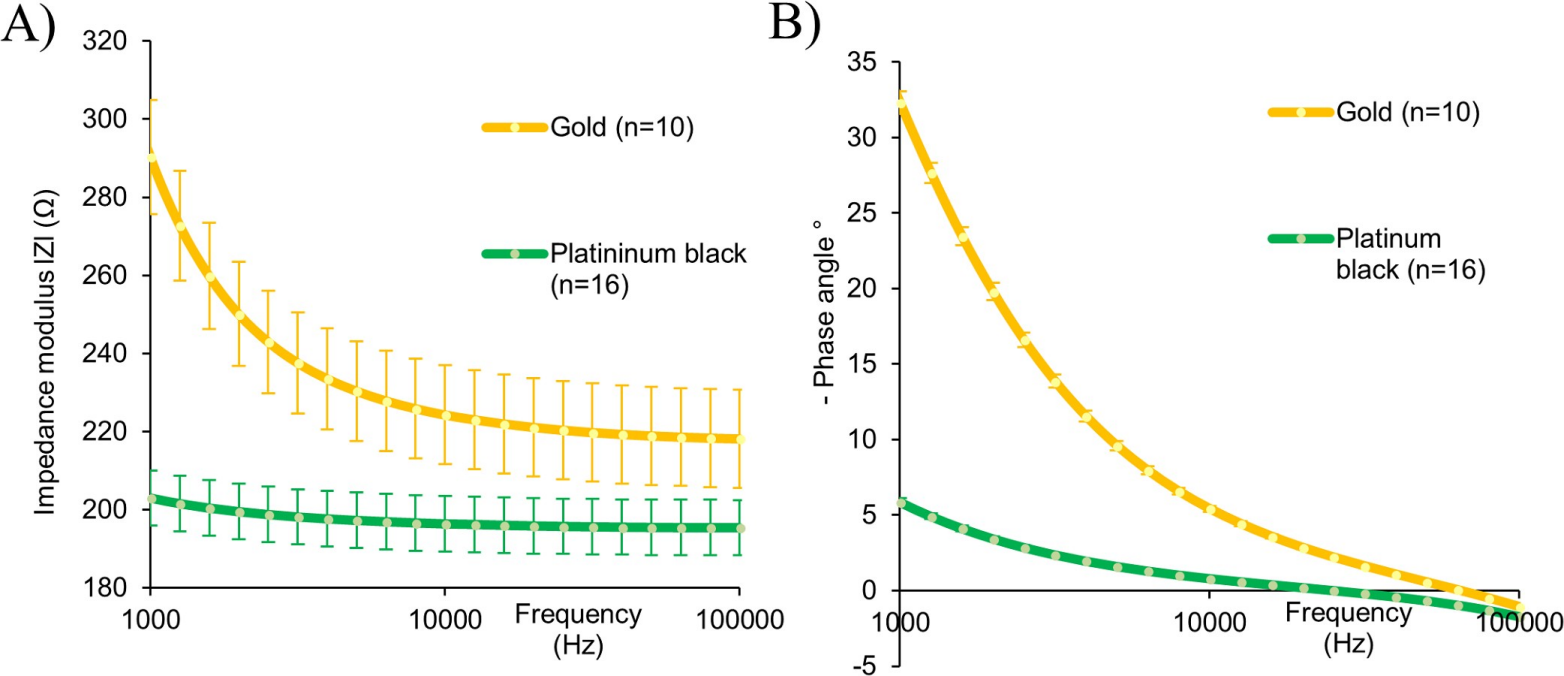
Impedance spectra of gold and platinised electrodes. (A) Impedance modulus and (B) phase angle. Data captured in EC media and represent the mean ± SEM (gold n = 10, platinum n = 16). Error bars are not visible for phase measurements at the scale used, max gold SEM 0.771° and max platinum black SEM 1.076°.

Impedance measurements were taken of bare gold electrodes in EC media before and after the addition of the thin film platinum black coating. Fig 3 shows that the addition of the platinum black layer coating achieved a reduction in total impedance (|Z|) and phase angle when compared to the gold substrate alone.

### Cell characterisation impedance measurements

A previously used method for monitoring cell monolayers using impedance is the total impedance normalised ratio, calculated by dividing the total impedance of a cell covered electrode at the beginning of the experiment with the total impedance of the same cell free electrode at the beginning of the experiment, defined in Eq 1 [21,35,42].

$$Total\;impedance\;normalised\;ratio=\frac{{\left|Z\right|}_{Cell\;covered\;electrode}}{{\left|Z\right|}_{Cell\;free\;electrode}}$$ (1)

**Fig 4:**
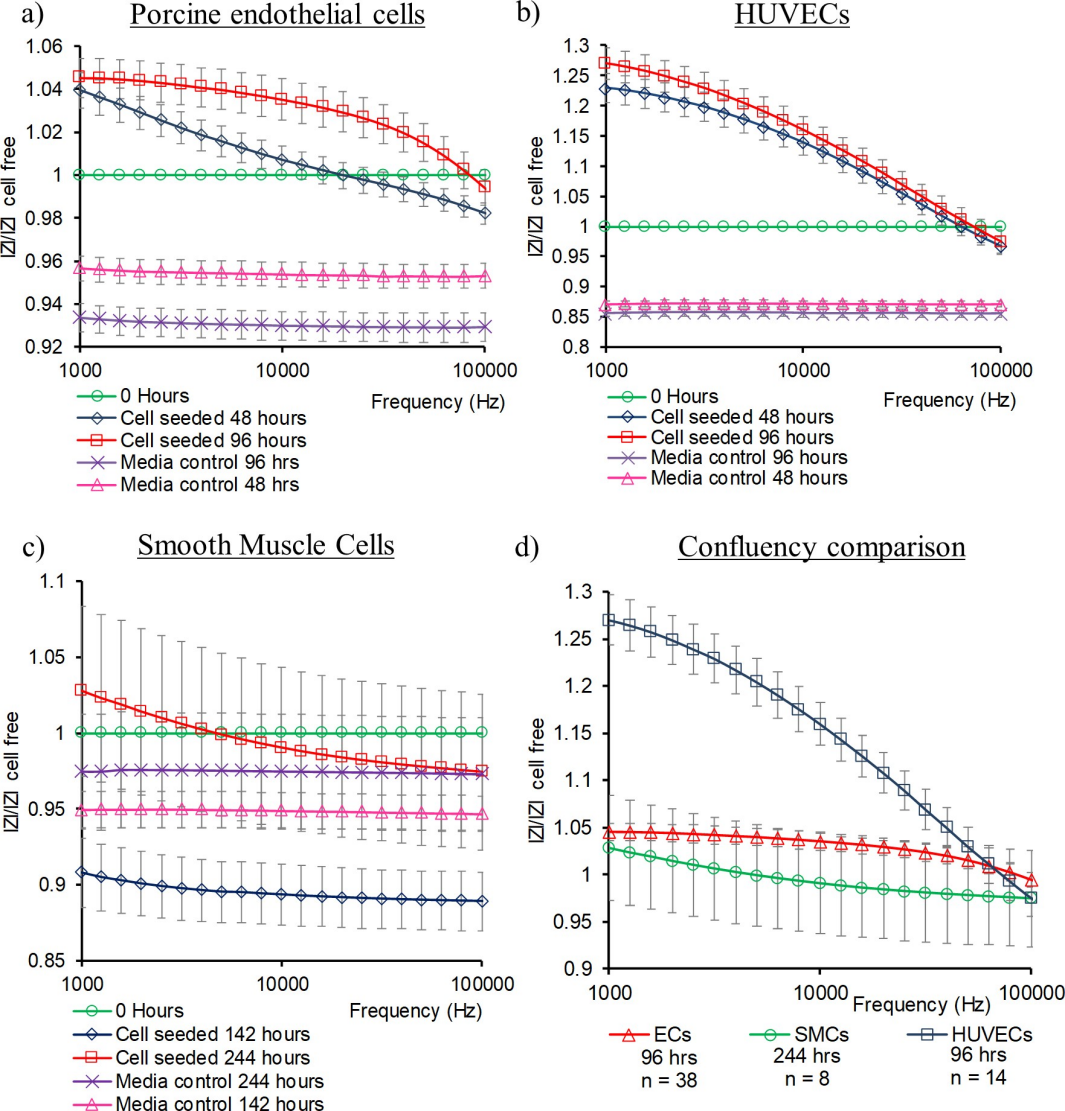
Mean total impedance ratio for confluent monolayers. (A) porcine ECs (n = 38) and media control electrodes (n = 33), (B) HUVECs (n = 14) and media control electrodes (n = 10), (C) SMCs (n = 8), and media control electrodes (n = 8), (D) Cell comparison at confluence. Error bars are ± SEM.

Mean electrode data using this ratio from the three cell types tested is displayed in Fig 4 along with the same ratio for media only cell free control electrodes. Porcine ECs (Fig 4A) and HUVECs (Fig 4B) displayed a similar trend of elevating values as confluence increased, with HUVECs exhibiting greater magnitudes. SMCs showed an initial decline in total impedance ratio that climbed towards cell free electrode values as they attained confluence, however cell seeded electrodes did not show large deviations from cell free control electrodes (Fig 4C).

**Fig 5:**
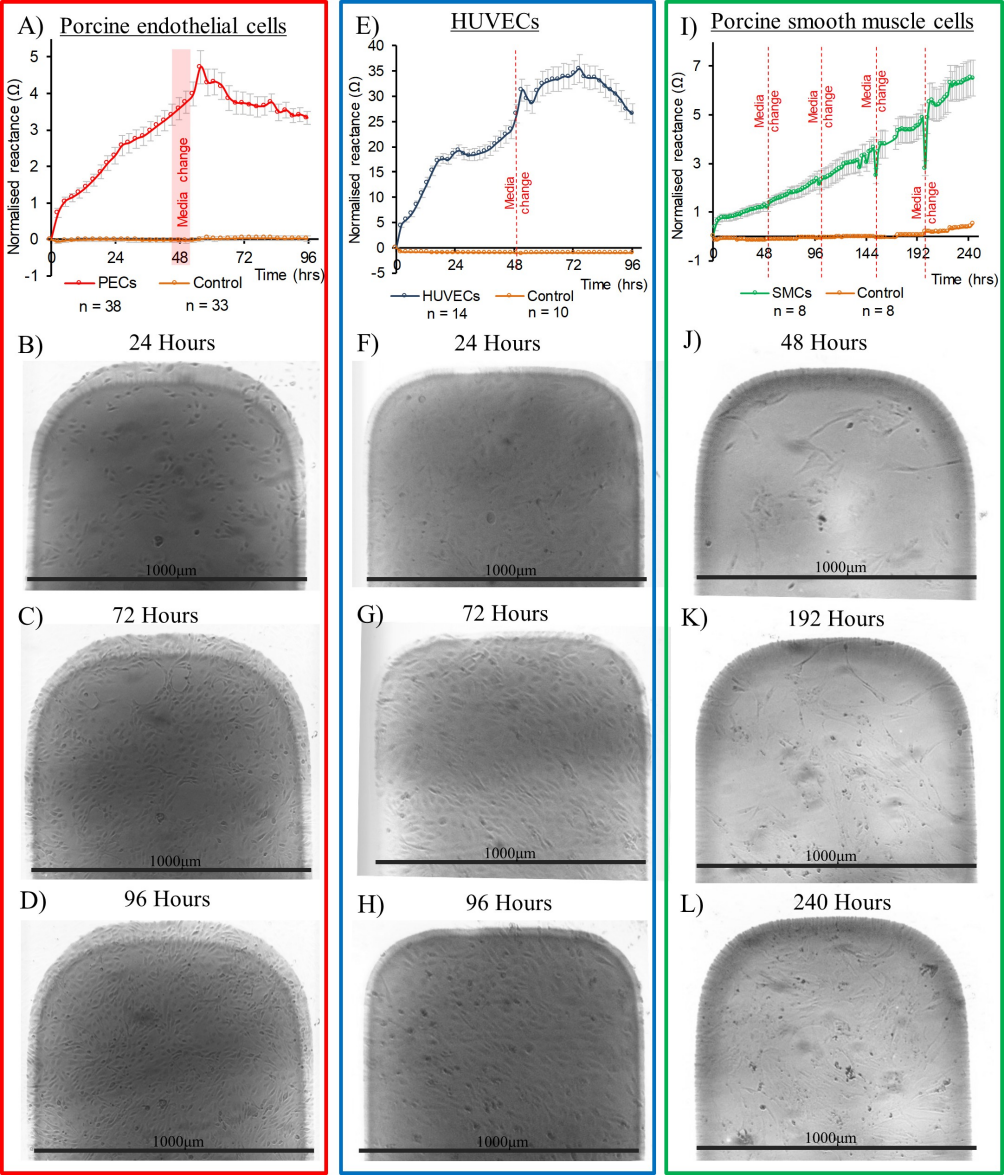
Reactance profiles at 10kHz and corresponding representative light microscopy images. Light microscopy images for Porcine ECs (B) to (D), HUVECs (F) to (H) and SMCs (J) to (L). Black scale bars are provided by known electrode width of 1000μm. Mean reactance profiles at 10kHz for cell seeded and cell free electrodes (A) Porcine ECs, (E) HUVECs and (I) SMCs. Red lines represent media changes. Error bars are ± SEM.

The capacitive reactance component of impedance has previously been used to monitor cell proliferation over electrodes [27,36,43,44]. Reactance data at electrodes seeded with the 3 vascular cell types is displayed in Fig 5, at the frequency of 10kHz, the median of the frequency range used in this study and a commonly used frequency for analysis in cellular impedance studies [31,34,45,46]. To highlight impedance variation arising from the effect of cellular proliferation reactance values were normalised by subtraction from the first values recorded immediately after cell seeding or media addition. Normalisation of impedance values in this fashion enables comparison with data sets reported in other impedance studies [34,47,48], and is also applied to measurements made using commercially available systems [31]. Also shown in Fig 5 are light microscopy images of representative electrode tips, showing cell proliferation to confluence. Microscopy observations showed that SMCs took longer to attain confluence than ECs, consistent with the slower rise in reactance observed for SMCs compared to ECs. All cell types exhibited a climb in reactance as cells first settled and then proliferated over the electrodes correlating with light microscopy observations. Minimal reactance variation was seen in control chambers containing media only. Following observation of confluence, ECs and HUVECs demonstrated a reduction in reactance magnitude (Fig 5A and 5E). Such a post confluence decline was not evident in the SMC data (Fig 5I). HUVECs demonstrated a similar reactance profile in comparison to ECs but with elevated magnitudes, 7 times higher at peak values (Fig 5E).

**Fig 6:**
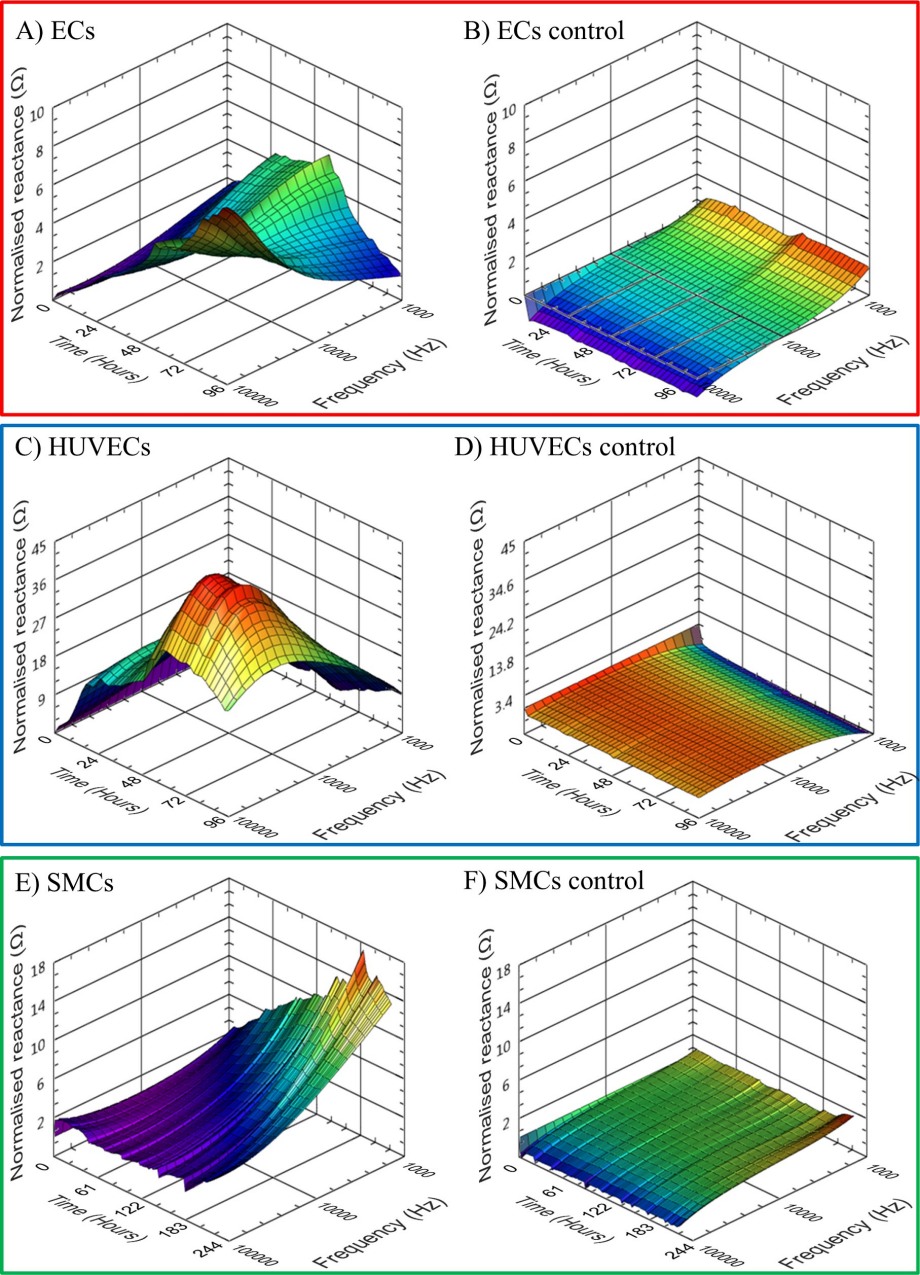
Mean electrode 3D normalised reactance profiles for duration of culture. (A) ECs n = 38, max SEM = 0.908, (B) EC control, n = 33, max SEM = 0.405, (C) HUVECs, n = 14, max SEM = 3.354, (D) HUVEC control, n = 10, max SEM = 1.054, (E) SMCs n = 8, max SEM = 1.861 and (F) SMC control, n = 8, max SEM = 0.778.

3D presentation of impedance variation in both time and frequency data has been demonstrated as an effective method to portray frequency dependent impedance data captured from cell monolayers [49–51]. Mean reactance data in the frequency range 1kHz to 100kHz, from the 3 vascular cell types is presented in Fig 6. ECs reactance displayed a complex pattern with peaks occurring earlier towards the lower 1kHz end of the spectrum and delayed at higher frequencies towards 100kHz (Fig 6A). HUVEC reactance was found to be maximal when measured at 30kHz after 72 hours of culture (Fig 6C). SMCs showed no reactance decreases at any of the measured frequencies (Fig 6E). Control electrodes are shown at the same scale as the corresponding cell covered electrodes and demonstrated minimal change in reactance over the measurement period (Fig 6B, 6D and 6F). 3D presentation of mean total impedance is available in supporting information S1 Fig.

## Discussion

The non-invasive monitoring of cellular regrowth in the post-stenting recovery phase has the potential to reveal insights into the progression of vascular diseases such as thrombosis, in-stent restenosis and neoatherosclerosis. Impedance spectroscopy is increasingly being used to monitor various aspects of cell function, although its application within the study of vascular pathologies has been more limited. To our knowledge, it has not been applied to characterise the vascular cell types that play important roles in such pathologies. To enable label-free characterisation of vascular cells, we developed an in-vitro impedance spectroscopy system to carry out continuous automated measurements in populations comparable to those seen covering implanted stents.

The addition of a platinum black coating to the electrodes used in the system reduced their overall impedance and is comparable to published data for platinum black electrodes [52]. Such impedance reductions from plain metal thin film electrodes can be attributed to an increased surface area for current flow owing to the coatings increased roughness, and improve sensitivity to impedance changes arising from cellular adhesion [53,54].

The total impedance ratio, previously used to identify motility [42] and cell detachment [35], here revealed distinct profiles for the three cell types examined. The total impedance of a monolayer can be correlated with its ability to form a selective boundary between its apical and basolateral sides, also termed its barrier function [30,31,55]. It is the formation of junctions between cells that contribute to its barrier function. SMCs do not form monolayer barriers in-vivo and with none of the tight junction complexes seen in endothelial cells [56], resulting in a comparably lower total impedance ratio on confluence. In the context of total impedance measurements taken of neointimal growth, reduced values may indicate a partially covered implant surface or a prevalence of non-barrier forming cell types such as SMCs.

Greater distinction between the vascular cell types was observed through 3D representation of the reactance component of impedance. Each reactance profile for 3 cell types, HUVECs, porcine endothelial and smooth muscle cells exhibited different characteristics and provides a potential method of non-invasively characterising these cell types. The elevation in reactance seen in all cell types as they proliferate over the electrode surface corresponds with similar studies involving impedance spectroscopy measurements of cell monolayers, with frequency (f) dependent reactance (Z”) typically converted into capacitance (C) according to the equation C = 1/2πfZ″ [57]. The reactance of a cell is determined by the capacitive effect of the cell membrane and various organelles within [57,58]. The shape and arrangements of these organelles and membranes are matched to each cell’s specific functionality and it is therefore reasonable to assume that capacitive reactance measurements of these elements of the cell will reveal differences between cell types [26,59], as presented here. It is acknowledged that impedance measured across a frequency range will report results from different regions of a cell monolayer, with the current path at low frequencies bypassing the cell membrane and measuring the paracellular space whilst higher frequencies are transcellular, through the cells interior [43,58,60,61]. By measuring over a frequency range that includes elements of paracellular and transcellular current pathways, the results presented here capture a spectrum of data throughout the different cell type monolayers that enables characteristic reactance profiles to be elucidated. The observed variation in reactance peaks between porcine aortic ECs and HUVECs provides evidence that the technique may be sufficiently sensitive to provide a means of characterisation between cell phenotypes, additional experimentation, with a wider variety of cell types, is required to further validate this proposal.

Post confluence declines in reactance were measured in the EC and HUVEC data with maximal falls of 78% and 32% respectively. However, these large reactance declines occurred with no corresponding change in microscopy observed cell morphology or cell death events. Total impedance and capacitive reactance reductions have been previously reported to correspond with induced cell death from cytotoxic agonists [62] and confluence related contact inhibition [63]. Non-death related events too can cause significant changes such as cell junction related barrier function modulation [30,31] and linage differentiation [27]. The observed reactance decline in confluent endothelial cell populations could be attributable to cell monolayer maturation processes such as cytoskeletal bound junction formation [64] and extra cellular matrix deposition [65]. Further experimentation will be required to investigate these hypotheses.

Our results show that simplified in-vitro models of the post-stenting regrowth scenarios of re-endothelialisation and ISR can be non-invasively characterised using impedance spectroscopic techniques. In the envisaged in-vivo environment cell regrowth patterns will occur over complex stent electrode geometries alongside pulsatile blood flow and it may be necessary to develop an electrode–cell model to simplify and elucidate the key impedance markers of cell specific proliferation. Such a model may also be useful in enabling clinicians to interpret impedance data. The translation of this in-vitro experimentation towards a self-reporting stent for use in a clinical environment as previously proposed [18,19] is however a considerable technological challenge. The electrical circuitry miniaturisation needed to fit inside an artery without occluding blood flow and non-contact transmission of data without the need for percutaneous leads represents a step change in current implanted electrical device design. Current miniaturisation of impedance devices for implantation is best represented in the 13mm diameter device developed by Rodriguez et al [66]. The most immediate use for the technology is therefore likely to be in the research domain with the development of an in-vivo research tool to help provide greater insights from the animal models that are widely used in this research field [67]. Such a device could capture data, in real-time, of cardiovascular disease progression, providing insights that help inform the development of advanced stents in the future.

The disease states of restenosis, in stent thrombosis and the optimal re-endothelialisation process take place in a complex, in-vivo, 3D environment comprising interaction with multiple cell types and pulsatile blood flow, in contrast to the 2D static single cell culture conditions described here. Further study in physiological flow conditions are therefore required in order to fully assess the potential of this technology. Intriguingly, it has previously been shown that endothelial cells in flow conditions exhibit increases in transendothelial resistance [68], suggesting that the cell specific impedance data presented here may be accentuated in in-vivo conditions. A further consideration is that in an in-vivo environment both smooth muscle cells and endothelial cells may proliferate over the stent struts, a scenario not included in our experiments. A co-culture model of smooth muscle cells with an upper layer of endothelial cells [69,70] would reveal further insights into the capabilities of impedance spectroscopy to characterise post-stenting cell regrowth. An additional limitation of the present study is the use of primary porcine cells. Whilst pigs can be considered as a good model for human cardiovascular disease and are used in a large array of cardiovascular studies [8,71,72] greater clinical relevance could be obtained through the use of primary human, coronary artery cells.

In summary the results show that analysis of the reactance component of impedance, if captured over a sufficiently wide frequency range, can provide a method of comparatively characterising vascular cells. This affirms the non-invasive characterisation capabilities of impedance spectroscopy previously demonstrated for other cell types when determining stem cell differentiation. Future work will aim to further examine vascular cell impedance properties via the monitoring of their respective differentiation processes.

## Supporting information

S1 File Impedance data set. (XLSX)

S1 Fig Mean electrode 3D normalised total impedance profiles for duration of culture. (A) ECs n = 38, max SEM = 2.96, (B) EC control, n = 33, max SEM = 1.54, (C) HUVECs, n = 14, max SEM = 6.79, (D) HUVEC control, n = 10, max SEM = 4.99, (E) SMCs n = 8, max SEM = 26.53 and (F) SMC control, n = 8, max SEM = 9.65.
(TIF)
