# Supplementary figures and images for: Towards non-invasive characterisation of coronary stent re-endothelialisation – An in-vitro, electrical impedance study
Source: PLoS One. 2018 Nov 5;13(11):e0206758. doi: 10.1371/journal.pone.0206758 (PMC6218196; doi:10.1371/journal.pone.0206758)

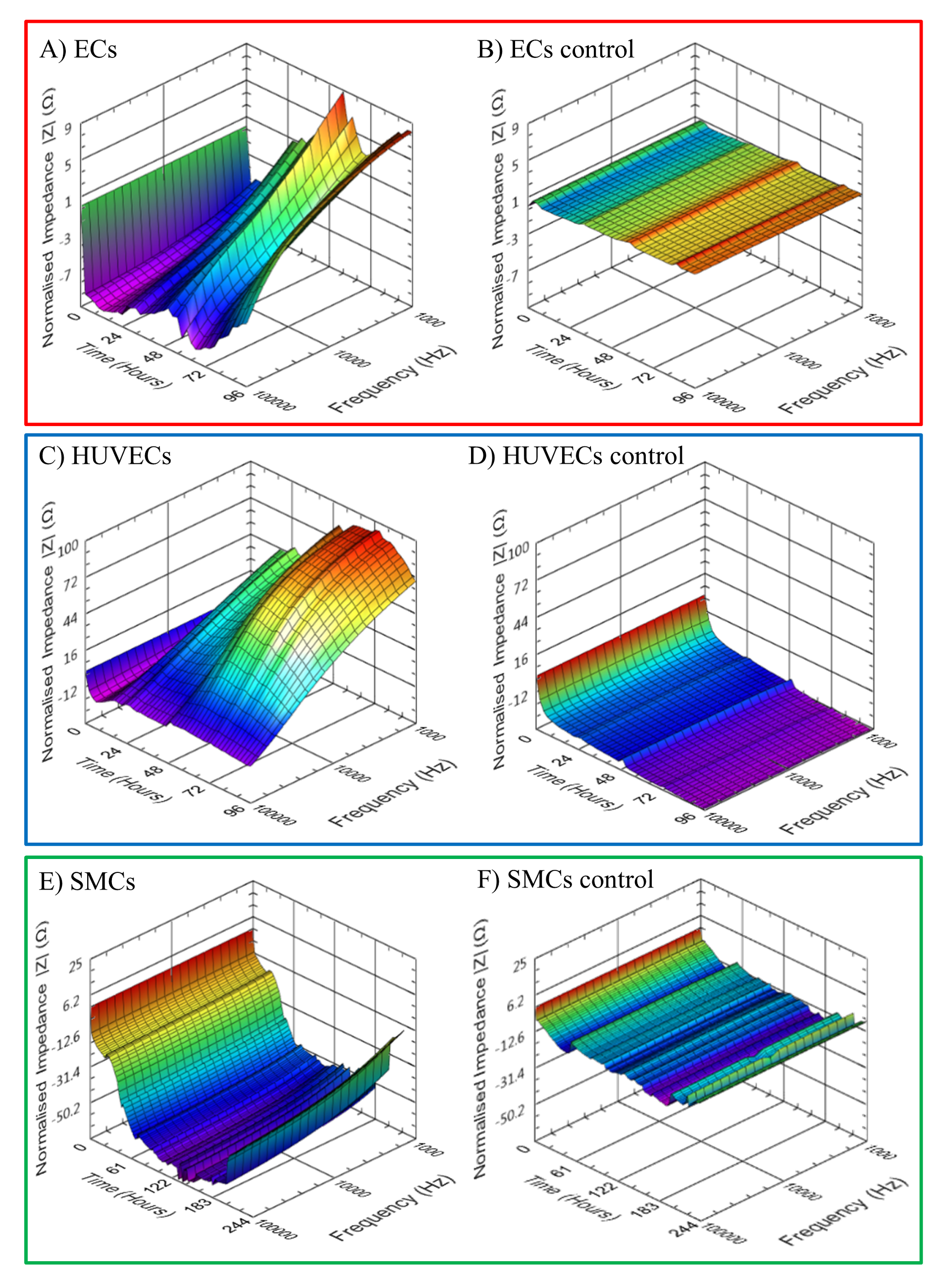

Supplement: S1 Fig — (A) ECs n = 38, max SEM = 2.96, (B) EC control, n = 33, max SEM = 1.54, (C) HUVECs, n = 14, max SEM = 6.79, (D) HUVEC control, n = 10, max SEM = 4.99, (E) SMCs n = 8, max SEM = 26.53 and (F) SMC control, n = 8, max SEM = 9.65. (TIF) [file pone.0206758.s002.tif]
